# The mediating role of motives in the associations between the big five personality traits and marijuana outcomes: A cross-national study across four countries

**DOI:** 10.1016/j.abrep.2026.100712

**Published:** 2026-05-27

**Authors:** Laura Mezquita, Adrian J. Bravo, Angelina Pilatti, Verónica Vidal-Arenas, Generós Ortet, Manuel I. Ibáñez, Cross-Cultural Addictions Study Team

**Affiliations:** a Department of Basic and Clinical Psychology and Psychobiology, Universitat Jaume I, Castelló de la Plana, Castellón, Spain; b Centre for Biomedical Research Network on Mental Health (CIBERSAM), Instituto de Salud Carlos III, Castellón de la Plana, Spain; c Department of Psychological Sciences, William & Mary, USA; d Universidad Nacional de Córdoba, Facultad de Psicología, Instituto de Investigaciones Psicológicas, IIPsi, CONICET. Córdoba, Argentina; e Department of Psychology and Sociology, Universidad de Zaragoza, Zaragoza, Teruel, Spain

**Keywords:** Personality, Big five, Marijuana motives, Marijuana, Cross-national study

## Abstract

**Background:**

Excessive marijuana use is associated with greater negative consequences, especially among youth. Identifying factors influencing these outcomes is crucial. While the Big Five personality traits are considered distal risk factors, marijuana motives act as more proximal mechanisms to marijuana use.

**Objectives:**

This study aimed to examine the mediating role of marijuana motives (Coping, Enhancement, Social, Expansion, and Conformity) in the associations between the Big Five personality traits and marijuana use frequency and negative consequences, and to test the generalizability of these results across college students from the U.S., Argentina, Spain, and the Netherlands.

**Method:**

A cross-sectional sample of 1031 university students who reported past-month marijuana use completed measures of the Big Five traits, marijuana motives, and marijuana-related negative consequences. Structural equation modeling was conducted to test the model and to test model invariance across countries.

**Results:**

In the whole sample, coping, expansion, and enhancement motives were significantly related to higher marijuana use frequency, while coping motives were significantly related to higher negative consequences. Furthermore, coping motives mediated the association of low emotional stability and marijuana outcomes, while expansion motives mediated the association of openness with marijuana frequency. Only minor differences arose across countries.

**Conclusions:**

The present findings suggest two risky personality pathways for marijuana use and misuse: a negative affect regulation pathway common to other drugs such as alcohol, and a second one related to openness to marijuana expansion effects. Personality-targeted interventions and prevention programs could benefit from additionally considering this last pathway in prevention/intervention efforts.

## Highlights

• Neuroticism is associated with greater marijuana use and related problems through coping motives*.*
• Openness is associated with greater marijuana use frequency through expansion motives.
• A negative affect regulation personality pathway explains marijuana use and problems.
• An openness-related expansion motives pathway is linked to marijuana use.
• Marijuana personality pathways generalize across four countries.

## 1 Introduction

Globally, marijuana is one of the most widely used psychoactive substances (World Health Organization, 2016) with an estimated 219 million users (4.3% of the global adult population) in 2021 (United Nations Office of Drugs and Crime [UNODC], 2023). From a public health concern, marijuana use is increasingly prevalent among youth, with 5.34% of the global youth population engaging in marijuana use (13.36% in North America, 7.04% in South America; 12.01% in Europe, UNODC, 2023). Excessive marijuana use is associated with poor health (Chandy et al., 2024) and with experiencing more negative marijuana-related consequences (Pearson, 2019), such as risky behaviors, academic/occupational consequences, or physical dependence in youths (Bravo, Pearson, et al., 2019). Moreover, among lifetime marijuana users, the probability of transitioning to marijuana use disorder is approximately 27% (Feingold et al., 2020). Therefore, identifying factors that influence marijuana use and its negative consequences is crucial for developing prevention and treatment programs.

From an integrative perspective, the Motivational Model of Substance Use (Cooper et al., 2016) provides a useful framework for understanding how biopsychosocial variables contribute to substance use. According to this model, relatively stable individual characteristics, such as personality traits, operate as distal factors that are indirectly related to substance use through more proximal determinants, namely motives for use. These motives are considered one of the most immediate predictors of substance use behaviors and related outcomes. Thus, this framework offers a coherent structure for integrating research on personality, marijuana use motives, and marijuana-related outcomes, and supports the examination of motivational processes as key mechanisms linking personality to marijuana use and misuse.

### 1.1 Personality and marijuana outcomes

The Five-Factor Model (FFM), also known as the Big Five, is currently the most widely used personality model worldwide (John, 2021). The model describes five personality traits labeled emotional stability (or low neuroticism), extraversion, openness, agreeableness and conscientiousness. Each of these traits has been associated with a range of health outcomes, including substance use (Soto, 2019). Previous meta-analyses have shown that high neuroticism, low conscientiousness and low agreeableness are related to substance use disorders (Kotov et al., 2010; Lui et al., 2022), while openness is additionality related to problematic marijuana use (Jain et al., 2025; Winters et al., 2022). Additionally, higher openness and lower agreeableness and conscientiousness are related to more frequent or intense marijuana use (Terracciano et al., 2008). Despite these well-established associations between the FFM and marijuana use and related problems, examining its associations with potential mediators, such as marijuana use motives, may help clarify the mechanisms through which personality relates to marijuana-related behaviors and inform more effective interventions.

### 1.2 Marijuana motives and marijuana outcomes

Marijuana motives are described as reasons why people use marijuana (Cooper et al., 2016). Building on the drinking motives literature (Cooper, 1994; Cooper et al., 1992), Simons et al. (1998) proposed a five-factor model of marijuana motives. According to this model, individuals may use marijuana to cope with distress (coping motives), enhance positive emotions (enhancement motives), facilitate social interactions (social motives), fit in with a group (conformity motives), or expand awareness and self-understanding (expansion motives). A meta-analysis indicated that each marijuana motive is differentially associated with marijuana outcomes (Bresin & Mekawi, 2019). Specifically, although all five motives were significantly and positively associated with marijuana-related problems, all motives except conformity were also linked to marijuana use frequency. However, after controlling for overlap among motives, only internal motives (i.e., enhancement, coping, and to a lesser extent, expansion) were positively related to marijuana use frequency, whereas conformity motives showed a small but significant negative association (Bresin & Mekawi, 2019). Furthermore, when controlling for the other motives, negative reinforcement motives (i.e., coping and conformity) were both significantly and positively related to marijuana-related problems, although the association was much stronger for coping motives than for conformity motives (Bresin & Mekawi, 2019).

### 1.3 Personality and marijuana motives

The association between personality and marijuana motives has been mainly explored using Conrod et al.'s (2000) substance abuse model, which differentiates four personality traits—anxiety sensitivity (AS), introversion/hopelessness (I/H), sensation seeking (SS), and impulsivity (IMP)—that are linked to elevated risk for substance use and misuse. For instance, Hecimovic et al. (2014), in a sample of 177 adult marijuana users, found that I/H was associated with coping motives, SS was positively associated with expansion motives, and AS was associated with conformity motives. Previous research in adolescent samples (Comeau et al., 2001), young adult marijuana users (Zvolensky et al., 2009), and treatment-seeking cigarette smokers (Guillot et al., 2018) also found associations between AS and negative affectivity with conformity motives.

A more recent study examined the role of dark personality traits—primarily characterized by antagonism and low conscientiousness (Vize et al., 2018)— in marijuana use through non-specific drug use motives (Sagioglou & Greitemeyer, 2024). Specifically, the authors reported that enhancement motives—and, to a lesser extent, expansion and social motives (i.e., positive reinforcement motives)—mediated the associations of psychopathy and masochism with marijuana use (Sagioglou & Greitemeyer, 2024). While less researched, one prior study among a community sample of past-month marijuana users examined the associations between the FFM and marijuana use motives (Glodosky & Cuttler, 2020). Their results indicated that after controlling for panic attack history, frequency of past-month marijuana use, and the other personality traits, only neuroticism was related to internal marijuana motives. They also found that anxiety sensitivity mediated the association between neuroticism and coping motives (Glodosky & Cuttler, 2020). Taken together, these studies suggest that neuroticism-related traits, such as hopelessness and anxiety sensitivity, are the personality traits most consistently linked to negative reinforcement marijuana motives (i.e., coping and conformity), whereas associations between other risky personality traits and marijuana motives have been less consistent.

### 1.4 Present study

The aim of the present research was to explore the mediating effects of marijuana motives (i.e., coping, social, expansion, conformity and enhancement) in the associations between the Big Five personality traits and marijuana outcomes (i.e., frequency and negative consequences) in a sample composed of university students from four different countries (i.e., U.S., Argentina, Spain and the Netherlands). Based on prior marijuana literature, we hypothesized that low emotional stability (i.e., high neuroticism) would be associated with greater marijuana frequency/consequences via greater coping motivations. Despite the extensive literature on personality etiological pathways to alcohol use (Mezquita et al., 2021), and the fact that some of the subjective effects associated with alcohol and marijuana consumption overlap (e.g., euphoria or mood enhancement), it could be hypothesized that some of these pathways may also be present in marijuana use (e.g., a positive affect regulation pathway: extraversion➔enhancement➔marijuana frequency). However, due to the limited empirical evidence in the marijuana literature, these pathways were considered exploratory in the present study. In addition, we tested whether the model was country-specific or universal across countries (i.e., invariant or non-invariant across countries). Cross-national comparisons are relevant, especially when examining marijuana use among countries with differing marijuana policies, because legislation regarding the access to marijuana, or the acceptability of consumption across sites among others, might impact the magnitude of associations across personality, marijuana use motives, and marijuana outcomes.

## 2 Method

### 2.1 Participants and procedure

Participants were college students (*N* = 3482) recruited from eight universities spanning five countries (U.S., Spain, Argentina, Uruguay, and the Netherlands) to complete an online survey exploring risk and protective factors of marijuana outcomes (see Bravo, Pearson, et al., 2019). However, for the purpose of this study the analytic sample was limited to 1031 students (61.76% women) who reported last month marijuana use and completed measures of personality, marijuana motives, and marijuana frequency and negative consequences([U.S., *n* = 678; 64.14% female; Spain, *n* = 148; 54.05% female; Argentina, *n* = 142; 58.45% female; the Netherlands, *n* = 63; 61.90% female; for demographics see Bravo, Pearson, et al., 2019). As only 29 students from Uruguay met the inclusion criteria, their answers were not included in the analyses. Study procedures were approved by the institutional review boards (or the international equivalent) for each participating university.

### 2.2 Measures

#### 2.2.1 Personality traits

The Big Five Personality Trait Short Questionnaire (BFPTSQ, Morizot, 2014) and its Spanish version (Ortet et al., 2017) were used to assess five personality domains: openness, extraversion, emotional stability (or low neuroticism), agreeableness, and conscientiousness. The BFPTSQ has 50 items answered on a 5-point response scale from 0 (*totally disagree*) to 4 (*totally agree*). Previous studies using Multigroup ESEM and ESEM-within-CFA approaches, have generally supported the measurement invariance of the BFPTSQ across Spanish- and English-speaking participants (Mezquita et al., 2019).

#### 2.2.2 Marijuana motives

Past 30-day marijuana motives were measured using the Marijuana Motives Measure Short Form (MMM-SF; Simons et al., 1998). For Spanish-speaking students, the Spanish version was administered (Mezquita, Ruiz-Valero, et al., 2018). The MMM-SF has 15 items (3 per scale) that are answered on a 5-point response scale from 1 (*almost never/never*) to 5 (*almost always/always*). Items were averaged for each motive such that higher scores indicate greater endorsement of a specific motive. The measure demonstrated configural and metric invariance across the four countries in a previous study, which represents the minimum level of invariance required to meaningfully compare associations between constructs across groups (Bravo, Sotelo, et al., 2019).

#### 2.2.3 Marijuana use

Typical marijuana use frequency was assessed using the Marijuana Use Grid (MUG, Pearson & Marijuana Outcomes Study Team, 2026). Each weekday was divided into six 4-h blocks of time (12a-4a, 4a-8a, 8a-12p, etc), and students were asked to report at which times they used marijuana during a “typical week” in the past 30 days. We were able to calculate marijuana frequency (summing the total number of time blocks for which they reported use [ranges: 0–42]).

#### 2.2.4 Negative marijuana-related consequences

Problematic marijuana use was assessed using the Brief Marijuana Consequences Questionnaire (B-MACQ; Simons et al., 2012) or its Spanish version (Bravo, Pearson, et al., 2019), a 20 item dichotomously scored measure that assesses negative consequences associated with marijuana use over the past 30 days. Previous research supported the configural and scalar measurement invariance of the instrument across English- and Spanish-speaking countries (Bravo, Pearson, et al., 2019). The total score (range 0–20) reflects the total number of consequences that the individual has experienced in the past month (see Table 1 for means and standard deviations by grouping).

**Table 1:** Descriptive analysis.

	USA^a^ *n* = 678		Argentina ^b^ *n* = 142		Spain ^c^ *n* = 148		The Netherlands ^d^ *n* = 63		a-b	a-c	a-d	b-c	b-d	c-d	Total sample *N* = 1031		
	M	SD	M	SD	M	SD	M	SD	*d*	*d*	*d*	*d*	*d*	*d*	M	SD	α
Age	19.52	2.57	24.67	4.58	21.21	3.53	20.78	2.43	−1.39	−0.55	−0.50	0.85	1.06	0.14	20.56	3.53	–
**Big 5 traits**																	
Emotional Stability	3.75	0.62	4.02	0.64	3.80	0.67	3.80	0.69	−0.43	−0.08	−0.08	0.34	0.33	0	3.80	0.64	0.85
Extraversion	3.61	0.76	3.57	0.72	3.61	0.69	3.69	0.70	0.05	0	−0.11	−0.06	−0.17	−0.12	3.61	0.74	0.86
Openness	3.55	0.57	3.76	0.51	3.62	0.58	3.57	0.66	−0.39	−0.12	−0.03	0.26	0.32	0.08	3.59	0.57	0.81
Agreeableness	3.37	0.60	3.23	0.53	3.09	0.60	3.16	0.66	0.25	0.47	0.33	0.25	0.12	−0.11	3.30	0.60	0.73
Conscientiousness	2.91	0.76	3.03	0.79	3.04	0.79	2.93	0.89	−0.15	−0.17	−0.02	−0.01	0.12	0.13	2.95	0.78	0.76
**Motives**																	
Social	2.57	1.22	2.49	1.17	2.52	1.11	2.26	1.13	0.07	0.04	0.26	−0.03	0.20	0.23	2.53	1.19	0.86
Coping	2.29	1.24	1.94	1.01	2.20	1.23	1.88	1.09	0.31	0.07	0.35	−0.23	0.06	0.28	2.21	1.20	0.89
Enhancement	3.83	1.14	3.43	0.96	3.34	1.14	3.79	1.13	0.38	0.43	0.04	0.09	−0.34	−0.40	3.70	1.13	0.79
Conformity	1.45	0.87	1.22	0.56	1.21	0.59	1.28	0.51	0.31	0.32	0.24	0.02	−0.11	−0.13	1.37	0.78	0.89
Expansion	2.48	1.33	2.64	1.20	2.09	1.08	2.14	1.27	−0.13	0.32	0.26	0.48	0.40	−0.04	2.43	1.29	0.89
**Marijuana Outcomes**																	
Marijuana Frequency	6.59	8.56	6.27	6.42	4.67	6.03	3.33	4.51	0.04	0.26	0.48	0.26	0.53	0.25	6.01	7.82	–
Negative Consequences	0.18	0.20	0.18	0.17	0.22	0.20	0.17	0.17	0	−0.20	0.05	−0.22	0.06	0.27	0.18	0.20	0.86

*Note*. Cohen's *d* values of 0.20, 0.50 and 0.80 correspond to small, medium and large effect sizes, respectively (Cohen, 1992).

### 2.3 Statistical analysis

Study aims were tested using a fully saturated path model (CFI = 1.00; TLI = 1.00; RSMEA = 0.000; SRMR = 0.000) using Mplus 8.4 (Muthén & Muthén, 2019), such that indirect effects were examined for the associations between Big Five personality traits and marijuana outcomes (i.e., marijuana frequency and marijuana negative consequences) via marijuana motives (e.g., emotional stability → coping → marijuana negative consequences). Age and gender were included as covariates within the model.

We examined the total, indirect, and direct effects of each predictor variable on marijuana outcomes using bias-corrected bootstrapped estimates (Efron & Tibshirani, 1993) based on 10,000 bootstrapped samples. This approach provides a powerful test of mediation (Fritz & MacKinnon, 2007) and one that is robust to small deviations from normality (Erceg-Hurn & Mirosevich, 2008). Statistical significance was determined by 99% bias-corrected bootstrapped confidence intervals that did not contain zero.

To test for structural invariance of the model across countries, we first conducted a freely estimated fully saturated multi-group model. Then, we conducted a constrained multi-group model where all paths in the model were constrained to equality across the four country groups. Within the constrained model, we tested which paths' liberation (i.e., freeing up the estimate across countries) resulted in the greatest contribution to improving model fit. Once we identified this path and allowed it to be freely estimated, we identified the next path with the greatest contribution at improving model fit. We repeated this procedure until χ^2^ was not significant at *p* > .01 (see Bravo, Sotelo, et al., 2019 for a similar analytic plan).

## 3 Results

For the total sample and across countries, Table 1 shows descriptive statistics and internal consistencies for all measures. All the internal consistencies were adequate, and Cohen's *d* (Cohen, 1992) showed that the differences in the mean levels of the scales among countries were relatively small. The only exception was the moderate difference between Argentina and the Netherlands on marijuana frequency (Argentina > the Netherlands).

Total, indirect, and direct effects for the mediation model are summarized in Table 2. The strongest direct effects of personality on motives were found between low emotional stability and low extraversion on coping motives, and between openness and expansion motives. Internal marijuana use motives showed significant and positive associations with marijuana frequency, and coping motives were additionally related to negative consequences. Focusing on indirect effects, low emotional stability and to a lesser extent introversion were related to marijuana use and marijuana negative consequences through coping motives, while openness and to a lesser extent introversion were related to marijuana use frequency through expansion motives.

**Table 2:** Summary of personality and motives effects on marijuana outcomes (M0).

Marijuana Outcome Variables:		*Marijuana Use*		*Negative Consequences*
**Predictor Variable:*****Emotional Stability***	**β**	**99% CI**	β	**99% CI**
Total	0.035	−0.058 0.124	−0.065	−0.149 0.024
Total indirect	−0.029	−0.073 0.011	**−0.054**	**−0.095 –0.020**
Social	0.000	−0.007 0.004	0.000	−0.007 0.004
Coping	**−0.038**	**−0.068 –0.016**	**−0.054**	**−0.092 –0.028**
Enhancement	−0.001	−0.015 0.011	−0.001	−0.011 0.008
Conformity	0.003	−0.006 0.018	−0.001	−0.015 0.003
Expansion	0.007	−0.014 0.030	0.001	−0.003 0.012
Direct	0.064	−0.025 0.152	−0.011	−0.096 0.076
**Predictor Variable:*****Extraversion***	**β**	**99% CI**	**β**	**99% CI**
Total	−0.011	−0.090 0.069	−0.037	−0.126 0.050
Total indirect	−0.026	−0.065 0.011	**−0.034**	**−0.065 –0.005**
Social	0.000	−0.003 0.009	0.000	−0.003 0.008
Coping	**−0.022**	**−0.045 –0.006**	**−0.031**	**−0.058 –0.010**
Enhancement	0.006	−0.004 0.023	0.004	−0.002 0.018
Conformity	0.008	0.000 0.024	−0.003	−0.016 0.006
Expansion	**−0.018**	**−0.041 –0.001**	−0.004	−0.017 0.003
Direct	0.015	−0.060 0.092	−0.003	−0.089 0.079
**Predictor Variable:*****Openness***	**β**	**99% CI**	**β**	**99% CI**
Total	0.015	−0.066 0.098	0.037	−0.055 0.124
Total indirect	**0.063**	**0.027 0.103**	0.022	−0.009 0.057
Social	0.000	−0.003 0.009	0.000	−0.003 0.008
Coping	0.009	−0.006 0.028	0.013	−0.008 0.037
Enhancement	0.006	−0.005 0.022	0.004	−0.003 0.017
Conformity	0.009	0.000 0.027	−0.003	−0.018 0.007
Expansion	**0.039**	**0.019 0.069**	0.008	−0.007 0.028
Direct	−0.048	−0.124 0.032	0.014	−0.078 0.103
**Predictor Variable:*****Agreeableness***	**β**	**99% CI**	**β**	**99% CI**
Total	−0.071	−0.141 0.001	−0.029	−0.107 0.052
Total indirect	−0.014	−0.050 0.021	−0.023	−0.052 0.004
Social	0.001	−0.006 0.013	0.001	−0.005 0.013
Coping	−0.013	−0.034 0.001	−0.018	−0.043 0.002
Enhancement	−0.003	−0.016 0.009	−0.002	−0.014 0.005
Conformity	0.009	−0.001 0.026	−0.003	−0.017 0.007
Expansion	−0.009	−0.031 0.011	−0.002	−0.013 0.002
Direct	−0.057	−0.124 0.011	−0.006	−0.081 0.071
**Predictor Variable:*****Conscientiousness***	**β**	**99% CI**	**β**	**99% CI**
Total	−0.026	−0.104 0.055	**−0.140**	**−0.220 –0.058**
Total indirect	0.002	−0.030 0.036	−0.002	−0.028 0.024
Social	0.000	−0.003 0.009	0.000	−0.003 0.009
Coping	−0.002	−0.018 0.013	−0.002	−0.025 0.018
Enhancement	0.000	−0.011 0.012	0.000	−0.008 0.009
Conformity	0.001	−0.008 0.013	0.000	−0.010 0.004
Expansion	0.002	−0.017 0.023	0.001	−0.004 0.009
Direct	−0.028	−0.101 0.047	**−0.138**	**−0.213 –0.060**
**Predictor Variable:*****Marijuana Motives***	**β**	**99% CI**	**β**	**99% CI**
Social	−0.016	−0.117 0.079	−0.019	−0.108 0.069
Coping	**0.174**	**0.083 0.264**	**0.249**	**0.156 0.344**
Enhancement	**0.117**	**0.035 0.197**	0.075	−0.014 0.160
Conformity	−0.095	−0.191 0.009	0.032	−0.071 0.141
Expansion	**0.208**	**0.114 0.299**	0.045	−0.040 0.128

*Note*. Significant associations are in bold typeface for emphasis and were determined bya 99% bias-corrected unstandardized bootstrapped confidence interval (based on 10,000 bootstrapped samples) that does not contain zero.

In testing for structural invariance, partial invariance was found, as seven paths had to be freely estimated to reach the benchmark (non-significant χ^2^ at *p* > .01) (see Table 3). Five of these paths were correlations between personality or motive variables, while the other two were: social motives ➔ marijuana use frequency and extraversion ➔ coping motives.

**Table 3:** Invariance testing results of the structural equation model across countries.

*Mediation Model Across Countries*														
			Overall Fit Indices							Comparison Fit Indices				
		χ^2^	*df*	p	CFI	TLI	RMSEA	SRMR	Model Comparison	Δχ^2^	Δ*df*	p	ΔCFI	ΔRMSEA
MG1	Full Constrained Model	415.73	270	0.0000	0.938	0.918	0.046	0.073	–	–	–	–	–	–
MG2	Full Constrained Model less Constraint 17	397.36	267	0.0000	0.945	0.926	0.044	0.072	MG1	18.378	3	0.0004	0.007	0.002
MG3	Full Constrained Model less Constraints 17, 62	379.98	264	0.0000	0.951	0.933	0.042	0.070	MG2	17.378	3	0.0006	0.006	0.002
MG4	Full Constrained Model less Constraints 17, 62, 52	363.23	261	0.0000	0.957	0.940	0.039	0.069	MG3	16.747	3	0.0008	0.006	0.003
MG5	Full Constrained Model less Constraints 17, 62, 52, 66	347.13	258	0.0002	0.962	0.947	0.037	0.066	MG4	16.097	3	0.0011	0.005	0.002
MG6	Full Constrained Model less Constraints 17, 62, 52, 66, 59	328.11	255	0.0014	0.969	0.956	0.034	0.062	MG5	19.029	3	0.0003	0.007	0.003
MG7	Full Constrained Model less Constraints 17, 62, 52, 66, 59, 28	313.85	252	0.0049	0.974	0.963	0.031	0.061	MG6	14.258	3	0.0026	0.005	0.003
MG8	Full Constrained Model less Constraints 17, 62, 52, 66, 59, 28, 63	302.00	249	0.0121	0.978	0.968	0.029	0.058	MG7	11.844	3	0.0079	0.004	0.002

*Note*. Constraint 17 refers to marijuana use on social motives (U.S.: β = 0.053 [−0.069 0.163], Arg.: β = 0.031 [−0.144 0.186], Sp: β = −0.170 [−0.387 0.078], Nth: β = −0.319 [−0.498–0.124]), 62 refers to coping with conformity (U.S.: *r* = 0.299 [0.197 0.397], Arg.: *r* = −0.032 [−0.252 0.209], Sp: *r* = 0.286 [0.081 0.482], Nth: *r* = 0.104 [−0.275 0.497]), 52 refers to extraversion with agreeableness (U.S.: *r* = 0.135 [0.034 0.234], Arg.: *r* = 0.257 [0.056 0.466], Sp: *r* = 0.447 [0.287 0.597], Nth: *r* = 0.217 [−0.056 0.473]), 66 refers to conformity with expansion (U.S.: *r* = 0.266 [0.161 0.363], Arg.: *r* = −0.006 [−0.247 0.262], Sp: *r* = 0.346 [0.136 0.526], Nth: *r* = 0.302 [−0.031 0.578]), 59 refers to social with conformity (U.S.: *r* = 0.407 [0.330 0.474], Arg.: *r* = 0.278 [0.095 0.418], Sp: *r* = 0.355 [0.187 0.498], Nth: *r* = 0.306 [0.048 0.544]), 28 refers to coping on extraversion (U.S.: β = −0.174 [−0.271–0.078], Arg.: β = 0.112 [−0.090 0.320], Sp: β = −0.124 [−0.293 0.046], Nth: β = 0.029 [−0.234 0.295]) and 63 refers to coping with expansion (U.S.: *r* = 0.405 [0.314 0.493], Arg.: *r* = 0.238 [0.034 0.420], Sp: *r* = 0.411 [0.228 0.574], Nth: *r* = 0.596 [0.364 0.773]).

Specifically, social motives were not significantly related to marijuana frequency in the U.S., Argentina and Spain, but it was significantly and negatively related to marijuana use frequency in the Netherlands. In addition, low extraversion was significantly related to coping motives in the U.S. but not in the other countries (see Table 3). Thus, Table 4 presents the indirect effects of personality on marijuana outcomes involved in these paths. The only path that showed a different pattern of association across countries was extraversion ➔ coping motives ➔ marijuana use / negative consequences, showing a significant indirect association between extraversion and marijuana outcomes via coping motives only in the U.S.

**Table 4:** Indirect effects of Personality on Marijuana outcomes through social and coping motives in U.S., Argentina, Spain and the Netherlands (MG6).

	U.S.		Argentina		Spain		the Netherlands	
	β	99% CI	β	99% CI	β	99% CI	β	99% CI
**Outcome:*****Marijuana Use***								
*Emotional Stability ➔ social*	0.000	−0.011 0.006	0.000	−0.014 0.007	0.001	−0.019 0.030	0.002	−0.032 0.038
*Extraversion ➔ social*	−0.001	−0.013 0.005	0.000	−0.014 0.006	0.002	−0.013 0.027	0.003	−0.022 0.034
*Openness ➔ social*	−0.001	−0.013 0.005	0.000	−0.014 0.006	0.002	−0.015 0.032	0.004	−0.027 0.038
*Agreeableness ➔ social*	−0.004	−0.020 0.004	−0.002	−0.021 0.011	0.012	−0.004 0.052	0.025	−0.005 0.068
*Conscientiousness ➔ social*	−0.001	−0.014 0.004	−0.001	−0.017 0.005	0.004	−0.012 0.031	0.008	−0.024 0.045
*Extraversion ➔ coping*	**−0.025**	**−0.051 –0.010**	0.017	−0.012 0.061	−0.028	−0.082 0.006	0.007	−0.062 0.072
**Outcome:*****Negative consequences***								
*Extraversion ➔ coping*	**−0.042**	**−0.076 0.019**	0.025	−0.021 0.080	−0.034	−0.095 0.010	0.008	−0.069 0.077

*Note.* Significant associations are in bold typeface for emphasis and were determined by a 99% bias-corrected unstandardized bootstrapped confidence interval (based on 10,000 bootstrapped samples) that does not contain zero.

## 4 Discussion

The present study tested the indirect associations between the Big Five traits of personality and marijuana outcomes (frequency and negative consequences) via marijuana motives in a cross-national sample of college students that reported past-month marijuana use. We further tested the model's invariance and generalizability across youth from the U.S., Argentina, Spain, and the Netherlands.

Our results showed that coping, expansion and enhancement marijuana motives were related to marijuana use frequency, and that coping motives were additionally related to negative consequences. These findings align with a meta-analysis which reported that, once the covariance between motives was controlled for, only internal marijuana motives (i.e., coping, expansion and enhancement) were significantly positively related to marijuana use, while coping motives were additionally and positively related to marijuana related problems (Bresin & Mekawi, 2019).

The only discrepancy with this previous meta-analysis was that conformity motives were not related to marijuana outcomes once the covariances between the other motives were controlled for. This difference might be due to our model including additional control variables. For example, both conformity motives (Comeau et al., 2001; Guillot et al., 2018; Hecimovic et al., 2014; Zvolensky et al., 2009) and marijuana-related problems (Winters et al., 2022) have been linked to traits associated with low emotional stability (e.g., neuroticism, anxiety sensitivity). Therefore, controlling for personality traits in our model may have accounted for this shared variance, reducing the unique contribution of conformity motives to marijuana outcomes. Moreover, these results together with previous research showing that the association between conformity motives and marijuana-related impairment is not consistently observed, also suggest that this relationship may be context-dependent and potentially moderated by other variables (e.g., race) (Buckner et al., 2015).

The path analysis with the whole sample also showed that low emotional stability, low conscientiousness and high openness were related to marijuana outcomes as in previous studies (Terracciano et al., 2008; Winters et al., 2022). However, while the association of conscientiousness was direct, the associations of emotional stability and openness were indirect through their associations with marijuana motives. Specifically, the indirect effects of low emotional stability on marijuana use and negative marijuana-related consequences through coping motives suggest a negative affect regulation pathway (or “self-medication” or “tension reduction” hypothesis), in which marijuana consumptions is driven to diminish negative affect states, like anxiety or depression. This pathway supports the relevance of a negative affect regulation pathway common to other psychoactive substances like alcohol (Mezquita, Bravo, et al., 2018; Mezquita et al., 2014), in which neuroticism-related traits play a prominent role.

Also, we found that openness was positively related to marijuana use frequency through expansion motives. This result suggests a substance-specific personality pathway for marijuana use, potentially due to the distinct effects of marijuana compared with other substances such as alcohol. Specifically, marijuana is strongly associated with alterations in perception, sensory experiences, and changes in cognitive processing (e.g., altered sense of time or enhanced awareness), whereas alcohol is typically linked to sedation and disinhibition***.*** That is, individuals that are imaginative, curious, and receptive to new ideas, feelings, and unconventional perspectives (i.e., open to the experience) may be more prone to use marijuana for seeking expanded experiential awareness (e.g., “to be more creative or original”, “to expand their awareness” or “to understand things differently”), which in turn would be related to a higher marijuana use frequency. This result also aligned with those found between dispositional mindfulness and marijuana use, as observing, a mindfulness dispositional trait closely related to openness (i.e., focus on perceptual experiences such as physical, visual, and auditory sensations), has been found to be related to higher endorsement of marijuana expansion motives, which in turn was associated with more severe symptoms of problematic marijuana use (Folivi et al., 2025).

Moreover, the fact that the main pathways showed a broadly similar pattern across countries suggests that the negative affect regulation and openness-to-expansion pathways may operate similarly among youths from the four countries involved in this study, despite differences in marijuana policy, social norms, and patterns of use.

Finally, although we found that low extraversion was significantly related to expansion and coping motives, the associations were weaker, and the invariance analyses showed that introversion related to marijuana outcomes through coping motives only in the U.S. Together with the fact that extraversion has not been consistently associated with marijuana use (Terracciano et al., 2008) or marijuana-related problems (Winters et al., 2022) in previous research, these findings suggest that the role of extraversion in personality pathways to marijuana use should be interpreted with caution when generalizing to other populations.

Overall, our results suggest a negative affect regulation pathway common to substance use and misuse in which low emotional stability personality traits are involved, while there are other etiological personality pathways (e.g., openness to expansion effects) that may be specific to marijuana use.

### 4.1 Limitations

Our results must be contextualized given the study's strengths and limitations. First, our data were cross-sectional, which precludes making causal inferences between variables. Thus, we could not test if changes in marijuana motives mediate the relationship between changes in personality and marijuana outcomes. Second, our sample was composed of university students and, therefore, may not generalize to the broader population of young adults. Third, although the students attended universities from four different countries, the selection of the participating universities was determined by researchers' contacts rather than for scientific purposes. Fourth, although there is a high endorsement of simultaneous use of alcohol and marijuana (SAM) among college students (Bravo et al., 2021), alcohol use was not controlled for in the present analyses; thus, future research should include measures of alcohol use, SAM, and specific motives linked to different patterns of use to more precisely disentangle overlapping influences. Finally, we focused on the association of personality and motives with marijuana outcomes. However, other variables have been previously related to marijuana consumption and marijuana-related problems (e.g., antisocial behavior, Solmi et al., 2021). Including these variables in more complex models would help us understand the complex phenomenon of marijuana use and misuse in a more integrated manner.

### 4.2 Conclusions

The results of this research highlight two different, but not mutually exclusive, personality etiological pathways to marijuana use and misuse. The negative affect regulation pathway is common not only to marijuana but also to other drug use and associated problems, such as those linked with alcohol (Mezquita, Bravo, et al., 2018; Mezquita et al., 2014) and suggests that prevention and intervention efforts may benefit from focusing on emotion regulation skills and reducing the use of substances as a coping strategy. In contrast, the openness-to-expansion pathway appears to be more specific to marijuana use and underscores the importance of targeting cognitive and perceptual expectancies associated with its use, as well as promoting alternative ways of exploring and enjoying unconventional experiences that do not involve marijuana use. Overall, these findings have important implications for prevention and clinical practice, as previous personality-targeted interventions for marijuana are often based on the same risk personality characteristics and motives for alcohol (e.g., Mahu et al., 2015).

## CRediT authorship contribution statement

**Laura Mezquita:** Writing – review & editing, Writing – original draft, Methodology, Funding acquisition, Formal analysis, Conceptualization. **Adrian J. Bravo:** Writing – review & editing, Project administration, Methodology, Funding acquisition, Data curation, Conceptualization. **Angelina Pilatti:** Writing – review & editing, Methodology, Funding acquisition, Conceptualization. **Verónica Vidal-Arenas:** Writing – review & editing, Methodology, Data curation. **Generós Ortet:** Writing – review & editing, Writing – original draft, Funding acquisition. **Manuel I. Ibáñez:** Writing – review & editing, Writing – original draft, Funding acquisition, Conceptualization.

## Funding

Dr. Bravo was supported by a training grant (T32-AA018108) from the 10.13039/100000027National Institute on Alcohol Abuse and Alcoholism (NIAAA) in the United States. Data collection was supported, in part, by grant T32-AA018108. NIAAA had no role in the study design, collection, analysis or interpretation of the data, writing the manuscript, or the decision to submit the paper for publication. Spanish authors received the following financial support for the research, authorship, and/or for the publication of this article: grant PID2022-141808OB-I00 from the MCIN/AEI/10.13039/501100011033/FEDER, UE; grant 2023I036 from the Government Delegation for the National Plan on Drugs; grant CIAICO/2024/47 from the Conselleria de Educación, Cultura y Universidades (Valencian Autonomous Government) and grant UJI-B2022–29 from the Universitat Jaume I. Data collection in Argentina was also supported by grants from the National Secretary of Science and Technology (FONCYT, grant number #PICT-2019-00457) and by grants from the 10.13039/501100008679Secretary of Science and Technology- National University of Córdoba (SECyT-UNC).

## Declaration of competing interest

The authors declare that they have no known competing financial interests or personal relationships that could have appeared to influence the work reported in this paper.

## Data Availability

All data, inputs, and outputs are available at https://osf.io/xeqdr
